# 15-Year outcome after normal exercise ^99m^Tc-sestamibi myocardial perfusion imaging: What is the duration of low risk after a normal scan?

**DOI:** 10.1007/s12350-012-9587-9

**Published:** 2012-06-08

**Authors:** Arend F. L. Schinkel, Henk J. Boiten, Jors N. van der Sijde, Pauline R. Ruitinga, Eric J. G. Sijbrands, Roelf Valkema, Ron T. van Domburg

**Affiliations:** 1 Department of Cardiology, Thoraxcenter, Erasmus Medical Center, Room Ba304, ‘s-Gravendijkwal 230, 3015 CE Rotterdam, The Netherlands; 2 Department of Internal Medicine, Section of Pharmacology, Vascular and Metabolic Diseases, Erasmus Medical Center, Rotterdam, The Netherlands; 3 Department of Nuclear Medicine, Erasmus Medical Center, Rotterdam, The Netherlands

**Keywords:** Coronary disease, prognosis, follow-up studies, radioisotopes

## Abstract

**Objective:**

The goal of this study was to evaluate the very long-term outcome after normal exercise ^99m^Tc-sestamibi myocardial perfusion single-photon emission computed tomography (SPECT). Exercise ^99m^Tc-sestamibi SPECT is widely used for risk stratification, but data on very long-term outcome after a normal test are scarce.

**Methods:**

A consecutive group of 233 patients (122 men, mean age 54 ± 12 years) with known or suspected coronary artery disease (CAD) underwent exercise ^99m^Tc-sestamibi SPECT and had normal myocardial perfusion at exercise and at rest. Follow-up endpoints were all-cause mortality, cardiac mortality, nonfatal myocardial infarction, and coronary revascularization. Predictors of outcome were identified by Cox proportional hazard regression models using clinical and exercise testing variables.

**Results:**

During a mean follow-up of 15.5 ± 4.9 years, 41 (18%) patients died, of which 13 were cardiac deaths. A total of 18 (8%) patients had a nonfatal myocardial infarction, and 47 (20%) had coronary revascularization. The annualized event rates for all-cause mortality, cardiac mortality, cardiac mortality/nonfatal infarction, and major adverse cardiac events were, respectively, 1.1%, 0.3%, 0.7%, and 1.8%. Multivariate analysis demonstrated that the variables age, male gender, diabetes, diastolic blood pressure at rest, rate pressure product at rest, peak exercise heart rate, and ST segment changes were independent predictors of major adverse cardiac events.

**Conclusion:**

Patients with suspected or known CAD and normal exercise ^99m^Tc-sestamibi myocardial perfusion SPECT have a favorable 15-year prognosis. Follow-up should be closer in patients with known CAD, and/or having clinical and exercise parameters indicating higher risk status.

## Introduction

Exercise ^99m^Tc-sestamibi myocardial perfusion single-photon emission computed tomography (SPECT) provides clinically useful information for diagnosis and risk stratification of patients with known or suspected coronary artery disease (CAD). Accurate risk stratification of these patients is required to optimize patient management. A recent meta-analysis of the literature demonstrated that normal myocardial perfusion SPECT has a high negative predictive value for cardiac events.^1^,^2^ Patients with normal myocardial perfusion SPECT are considered at low risk of cardiac events, the annualized event rate is generally <1% during the first few years after testing. Accordingly, in these low-risk patients, further (invasive) diagnostic and therapeutic strategies and associated medical care costs can be avoided.^1^-^3^

However, over time, a significant change in risk may occur after a normal myocardial perfusion SPECT. The underlying clinical risk and history of CAD significantly influence the event rate after a normal myocardial perfusion SPECT. Moreover, a temporal component of risk has been identified, which may increase the annualized cardiac event rate to 2%, even in the presence of a normal myocardial perfusion SPECT.^4^ These observations have led to the perception that a “warranty period” exists after a normal myocardial perfusion SPECT. In the currently available literature, mean follow-up after myocardial perfusion SPECT was approximately 3 years.^1^-^3^ Data of very long-term outcome after normal myocardial perfusion SPECT are lacking, and consequently, the duration of the low-risk status after a normal test is not clear. This creates uncertainties in patient management recommendations. The goals of the current study were as follows: (1) To assess very long-term outcome after normal myocardial perfusion SPECT. (2) To define a low-risk period after normal myocardial perfusion SPECT. (3) To identify predictors of increased risk.

## Methods

### Study Design

The study population consisted of 242 consecutive patients with known or suspected CAD who underwent exercise ^99m^Tc-sestamibi myocardial perfusion SPECT and had normal myocardial perfusion at exercise and at stress. The majority of the study population has been described in a previous study from our center.^5^ The local medical ethics committee approved the protocol, and all the patients gave informed consent. A structured interview and clinical history were obtained, including assessment of cardiac risk factors, and the symptoms before the exercise test.

### Exercise Testing Protocol

All the patients performed a symptom-limited upright bicycle ergometry test with stepwise increment of 20 W every minute. Cuff blood pressure measurement and standard 12-lead surface electrocardiograms were obtained at rest and every minute during exercise, until the end of the recovery phase. The electrocardiograms were digitally stored and analyzed by an experienced observer. Test endpoints included the following: severe angina, decrease in systolic blood pressure fall >40 mm Hg, blood pressure >240/120 mm Hg, or significant cardiac arrhythmia. An ischemic response was defined as ≥1 mm horizontal or downsloping ST-segment depression at 80 ms after the J point.

### Myocardial Perfusion SPECT

Approximately 1 minute before the termination of the exercise test, an intravenous dose of 370 MBq of ^99m^Tc-sestamibi was administered as previously described.^5^ For resting studies, 370 MBq of the same tracer was administered at least 24 h after the exercise test. Image acquisition was performed using a SPECT camera system (Orbiter camera; Siemens, Iselin, NJ; or Picker Prism 3000XP camera; Picker, Cleveland, OH). Thirty-two projections were obtained, from the left posterior oblique to the right anterior oblique over 180°. The semiquantitative interpretation of the scan was performed by visual analysis assisted by the circumferential profiles analysis. Stress and rest tomographic views were reviewed side-by-side by two experienced observers who were unaware of the patients’ clinical data. In case of disagreement, a majority decision was achieved by a third observer. A normal study was defined as the absence of perfusion abnormalities.

### Patient Follow-Up

Follow-up data were collected in the year 2011 and were completed in respect of 233 patients (96%). Outcome data were obtained by evaluation of hospital records, contacting the patient’s general practitioner, and/or review of civil registries. The date of the last review or consultation was used to calculate follow-up time. Endpoints were all-cause mortality, cardiac death, nonfatal myocardial infarction, and coronary revascularization. Nonfatal myocardial infarction was defined as new symptoms of ischemia, and/or ECG changes indicative of new ischemia, and/or imaging evidence of myocardial infarction, accompanied by detection of a rise and fall of cardiac biomarkers.^6^ Major adverse cardiac events (MACE) were defined as the occurrence of cardiac death, nonfatal myocardial infarction, or revascularization.

### Statistical Analysis

Values were expressed as mean ± SD or number, and compared using the Student’s *t* test or chi-squared test. Univariate and multivariate Cox proportional hazard regression models (SPSS statistical software version 15.0, SPSS, Chicago, IL) were used to identify independent predictors of outcome.^7^ Variables were selected in a stepwise forward selection manner with entry and retention set at a significance level of .05. The risk of a variable was expressed as a hazard ratio with a corresponding 95% confidence interval. The probability of survival was calculated using the Kaplan-Meier method, and survival curves were compared using the log-rank test. *P* value <.05 was considered statistically significant.

## Results

### Clinical Characteristics

Clinical characteristics of the 233 patients are summarized in Table 1. A total of 222 (95%) patients had an interpretable ECG at baseline. There were no major side effects or complications as result of the test. There was a significant increase in heart rate (78 ± 15 to 147 ± 24 beats/minute, *P* < .001), and systolic blood pressure (138 ± 22 to 188 ± 25 mm Hg, *P* < .001 from rest to peak exercise. The mean workload was 143 ± 43 W, corresponding with an exercise duration of 7 minutes. The target heart rate (85% of the maximal predicted heart rate) was reached in 173 patients (74%). Exercise-induced angina occurred in 29 (12%) patients, and 19 (8%) had ST segment depression during the exercise test.

**Table 1:** Patient characteristics

Age (years)	54 ± 12
Men	122 (52%)
Height (cm)	170 ± 10
Weight (kg)	74 ± 13
Hypertension	78 (33%)
Smoking	61 (26%)
Hypercholesterolemia	63 (27%)
Diabetes mellitus	16 (7%)
ACE-inhibitor	29 (12%)
Beta-blocker	78 (33%)
Known coronary artery disease	57 (24%)
Prior coronary revascularization	55 (24%)
Prior myocardial infarction	19 (8%)
Typical angina	58 (25%)
Atypical angina	120 (52%)
Nonspecific symptoms	14 (6%)
No symptoms	41 (18%)

### Outcome

**Figure 1:**
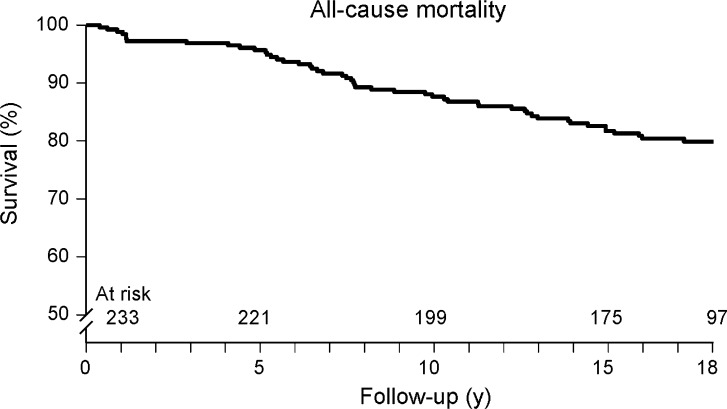
Kaplan-Meier event-free survival for the endpoint of all-cause mortality in patients with normal exercise ^99m^Tc-sestamibi myocardial perfusion SPECT

**Figure 2:**
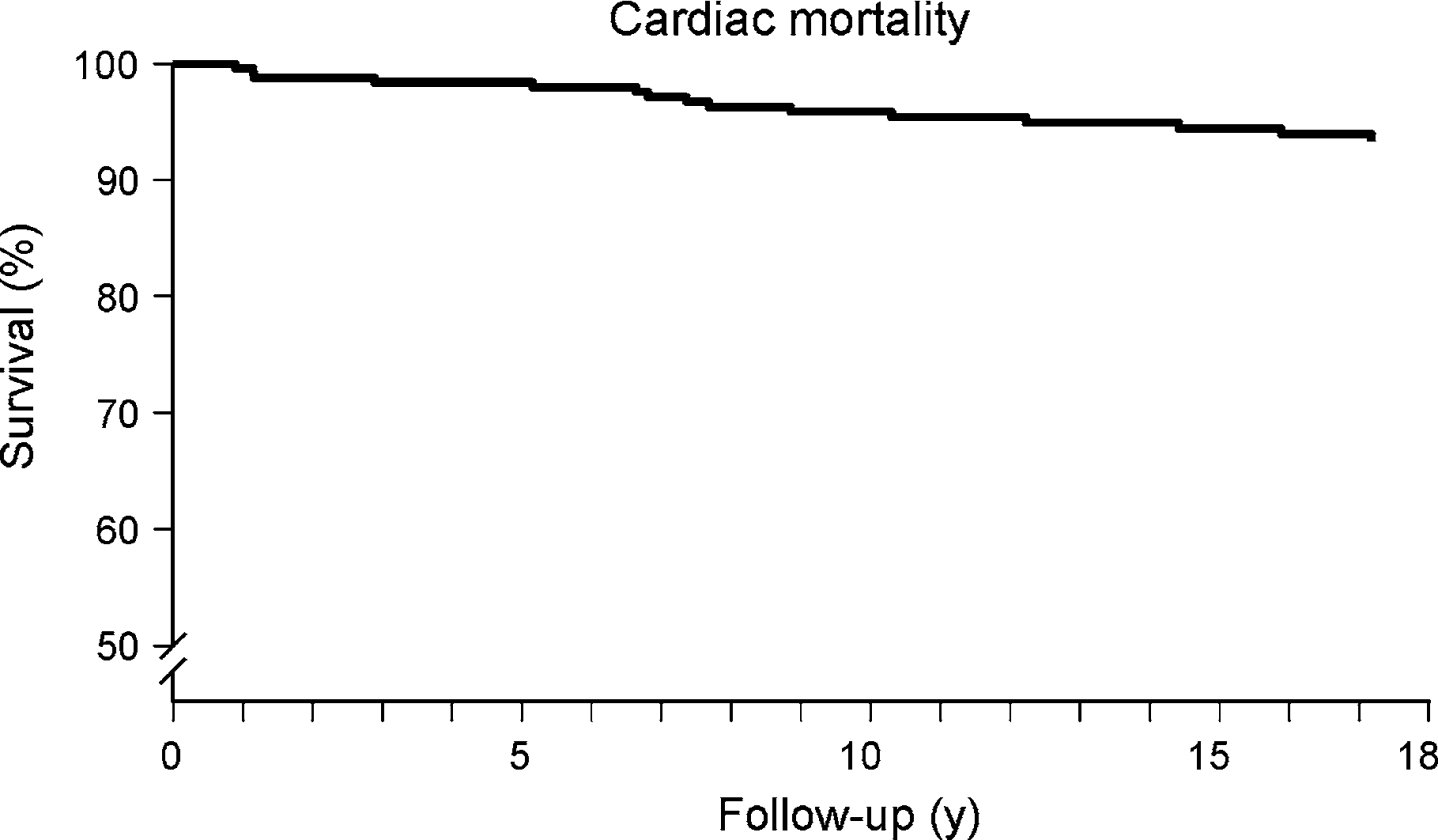
Kaplan-Meier event-free survival for the endpoint of cardiac mortality in patients with normal exercise ^99m^Tc-sestamibi myocardial perfusion SPECT

**Figure 3:**
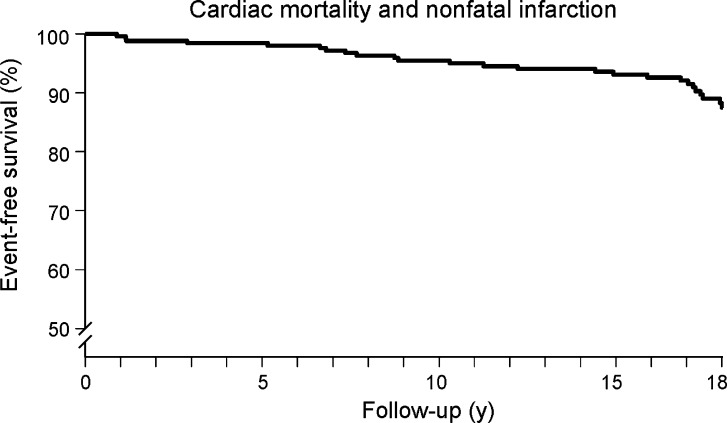
Kaplan-Meier event-free survival for the endpoint of cardiac mortality and nonfatal myocardial infarction in patients with normal exercise ^99m^Tc-sestamibi myocardial perfusion SPECT

**Figure 4:**
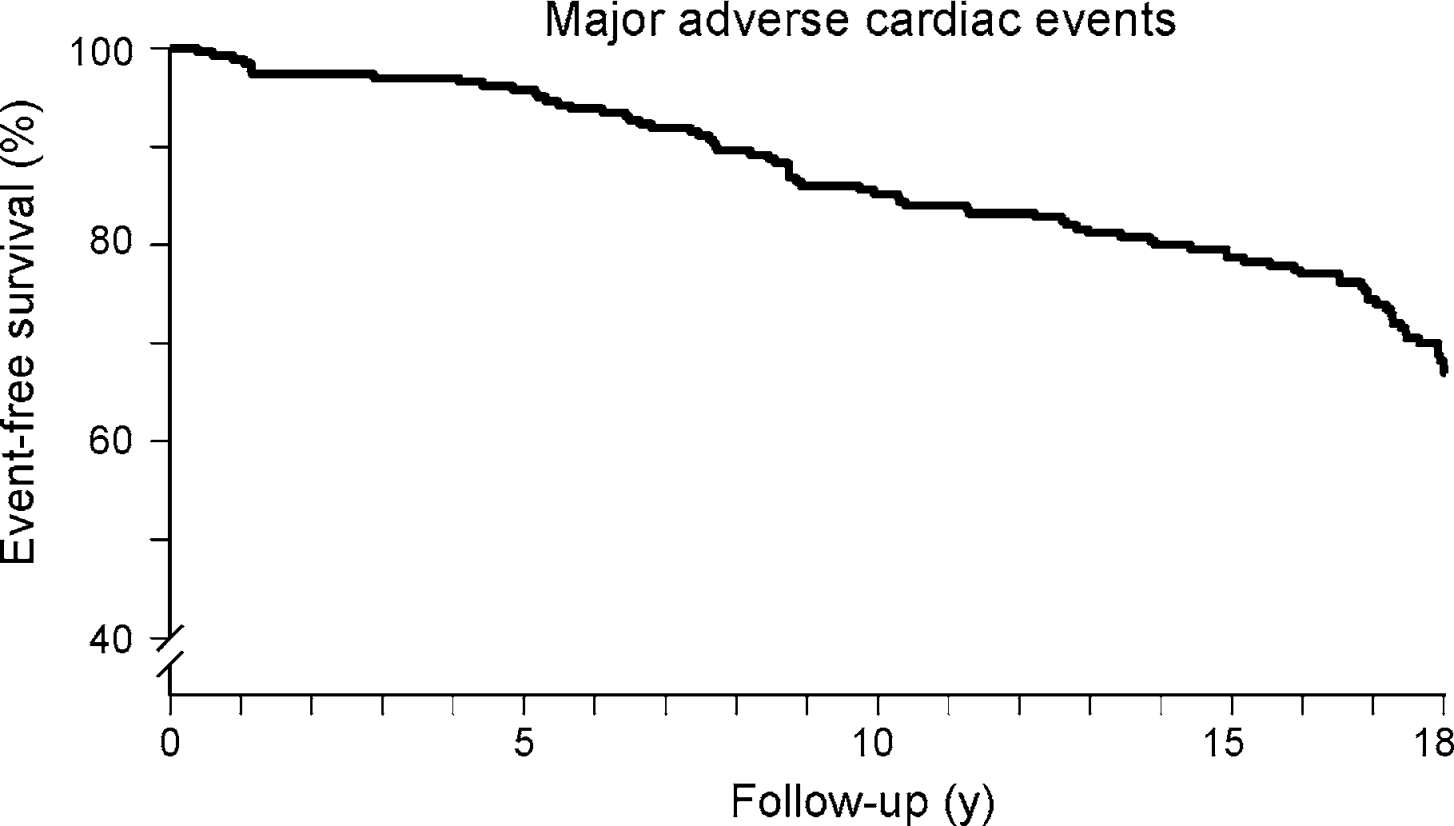
Kaplan-Meier event-free survival for the endpoint of major adverse cardiac events in patients with normal exercise ^99m^Tc-sestamibi myocardial perfusion SPECT

**Figure 5:**
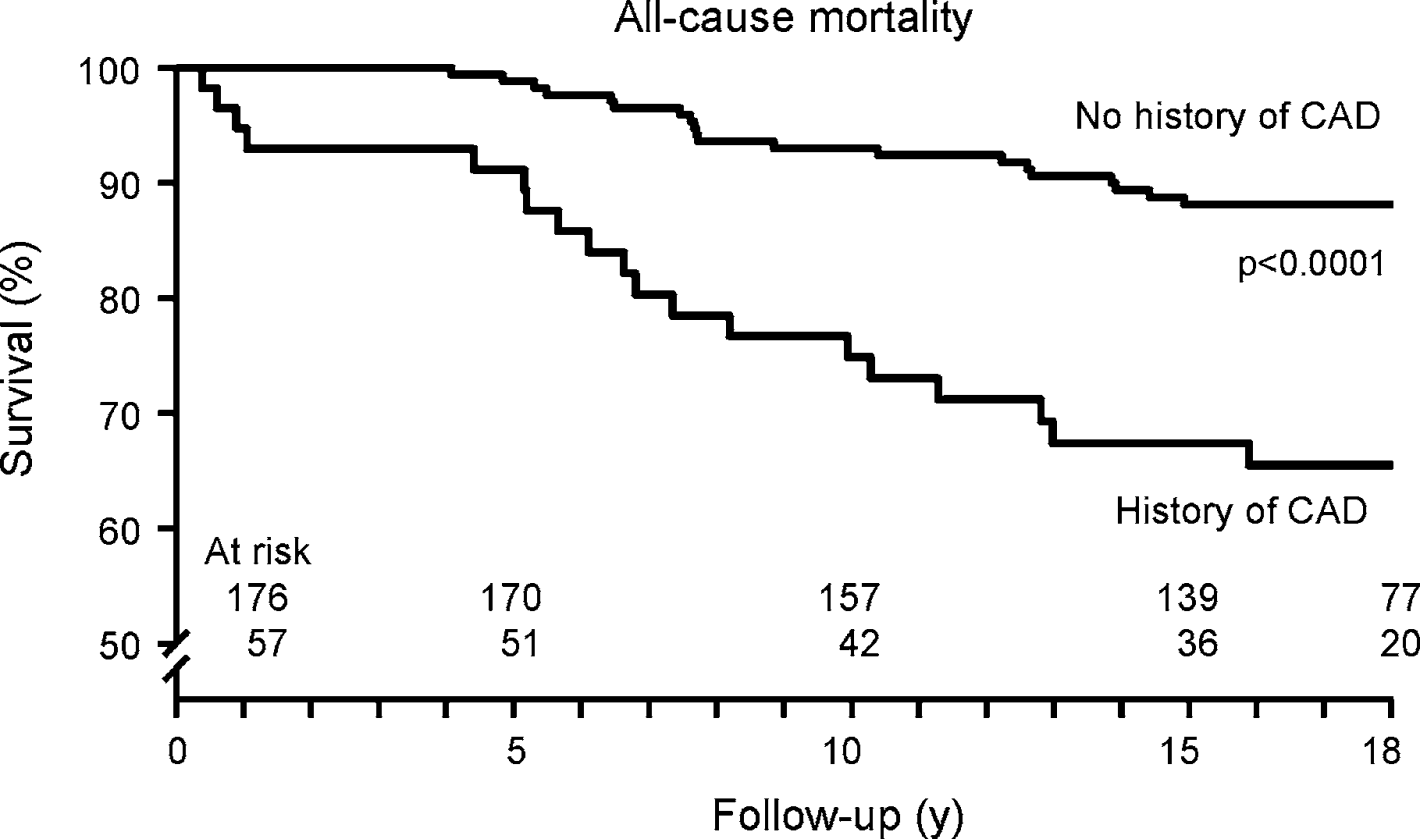
Kaplan-Meier event-free survival for the endpoint of all-cause mortality in patients with normal exercise ^99m^Tc-sestamibi myocardial perfusion SPECT, with or without a history of CAD

Kaplan-Meier survival curves and cumulative event rate are presented in Figures 1, 2, 3, 4, and 5. During a mean follow-up of 15.5 ± 4.9 years, 41 (18%) patients died, of which 13 were cardiac deaths. A total of 18 (8%) patients had a nonfatal myocardial infarction. Coronary revascularization procedures were performed in 47 patients (20%). Seventeen patients (7%) underwent coronary artery bypass surgery, and 30 (13%) underwent percutaneous coronary intervention. The annualized event rates for all-cause mortality, cardiac mortality, cardiac mortality/nonfatal infarction, and major adverse cardiac events were respectively 1.1%, 0.3%, 0.7%, and 1.8%. All-cause mortality was significantly higher in patients with known CAD (Figure 5).

### Risk Stratification

Univariate analysis demonstrated that age, diabetes mellitus, diastolic blood pressure at rest, and heart rate during exercise was predictors of all-cause mortality (Table 2). Diastolic blood pressures at rest and peak exercise heart rate were predictors of cardiac mortality. Beta-blocker use, and peak exercise heart rate were predictors of cardiac mortality/nonfatal infarction. Age, male gender, diastolic blood pressure at rest and peak exercise heart rate were predictors of major adverse cardiac events.

**Table 2:** Univariate predictors of outcome

	All-cause mortality	Cardiac mortality	Cardiac mortality/nonfatal infarction	Major adverse cardiac events
Clinical features				
Age >70 years	7.46 (2.84–19.51)	2.52 (0.51–12.56)	1.39 (0.38–5.10)	3.59 (1.37–9.38)
Male gender	1.53 (0.77–3.05)	2.53 (0.65–9.78)	1.96 (0.84–4.57)	2.44 (1.41–4.24)
Hypertension	0.79 (0.38–1.65)	0.43 (0.09–2.03)	1.43 (0.63–3.24)	1.43 (0.82–2.49)
Smoking	0.76 (0.34–1.69)	1.06 (0.27–4.13)	0.78 (0.30–2.05)	0.65 (0.34–1.21)
Hypercholesterolemia	0.60 (0.26–1.39)	0.26 (0.03–2.06)	1.16 (0.48–2.79)	1.19 (0.66–2.15)
Diabetes mellitus	3.12 (1.07–9.14)	1.38 (0.17–11.52)	2.81 (0.84–9.44)	2.35 (0.84–6.57)
ACE–inhibitor	1.97 (0.81–4.84)	0.69 (0.09–5.62)	0.53 (0.12–2.37)	1.25 (0.57–2.76)
Beta-blocker	0.91 (0.44–1.87)	1.70 (0.50–5.76)	2.39 (1.06–5.38)	1.62 (0.93–2.83)
Prior myocardial infarction	1.77 (0.60–5.21)	1.13 (0.14–9.37)	2.21 (0.68–7.24)	2.57 (0.99–6.66)
Stress test results				
Heart rate at rest	0.99 (0.89–1.18)	0.92 (0.61–1.40)	0.84 (0.64–1.11)	0.91 (0.79–1.04)
Peak exercise heart rate	0.80 (0.71–0.90)	0.60 (0.46–0.86)	0.72 (0.60–0.86)	0.82 (0.75–0.90)
Diastolic blood pressure rest	0.75 (0.58–0.97)	0.55 (0.31–0.98)	0.70 (0.46–1.05)	0.77 (0.63–0.95)
Rate pressure product at rest	1.03 (0.93–1.14)	0.96 (0.75–1.22)	0.94 (0.80–1.10)	0.97 (0.90–1.05)
Typical angina	0.72 (0.24–2.20)	0.69 (0.09–5.62)	1.26 (0.40–3.94)	2.02 (0.92–4.43)
ST segment changes	0.53 (0.12–2.38)	0.00 (0.00–4.18)	0.89 (0.19–4.08)	1.28 (0.49–3.31)

Values are expressed as Cox proportional hazard ratio and 95% confidence interval.

Multivariate models demonstrated that age, male gender, diabetes, heart rate at rest and peak exercise heart rate were independent predictors of all-cause mortality (Table 3). Heart rate at rest and peak exercise heart rate were predictors of cardiac mortality (Table 4). Male gender, diabetes, and peak exercise heart rate were predictors of cardiac mortality/nonfatal infarction (Table 5). Age, male gender, diabetes, diastolic blood pressure at rest, rate pressure product at rest, peak exercise heart rate, and ST segment changes were independent predictors of major adverse cardiac events (Table 6).

**Table 3:** Multivariate predictors of all-cause mortality

	HR (95% CI)	*P* value
Age*	1.06 (1.04–1.09)	<.001
Male gender	2.70 (1.45–5.03)	.002
Diabetes	3.06 (1.22–7.65)	.02
Heart rate at rest	1.30 (1.07–1.58)	.01
Peak exercise heart rate	0.80 (0.69–0.92)	.03

Values are expressed as Cox proportional hazard ratio and 95% confidence interval.

* Per 1 unit increment.

**Table 4:** Multivariate predictors of cardiac mortality

	HR (95% CI)	*P* value
Heart rate at rest	1.73 (1.02–2.94)	.04
Peak exercise heart rate	0.50 (0.35–0.69)	<.001

Values are expressed as Cox proportional hazard ratio and 95% confidence interval.

**Table 5:** Multivariate predictors of cardiac mortality/nonfatal myocardial infarction

	HR (95% CI)	*P* value
Male gender	2.61 (1.11–6.14)	.03
Diabetes	6.93 (2.18–22.04)	.01
Peak exercise heart rate	0.70 (0.58–0.84)	<.001

Values are expressed as Cox proportional hazard ratio and 95% confidence interval.

**Table 6:** Multivariate predictors of major adverse cardiac events

	HR (95% CI)	*P* value
Age*	1.03 (1.01-1.06)	.01
Male gender	2.82 (1.75-4.53)	<.001
Diabetes	3.95 (1.88-8.30)	<.001
Diastolic blood pressure at rest	0.79 (0.64-0.97)	.03
Rate-pressure product at rest	1.14 (1.04-1.25)	.01
Peak exercise heart rate	0.80 (0.71-0.89)	<.001
ST segment changes	2.94 (1.44-5.99)	.01

Values are expressed as Cox proportional hazard ratio and 95% confidence interval.

* Per 1 unit increment.

## Discussion

In this study, very long-term outcome after normal exercise ^99m^Tc-sestamibi myocardial perfusion SPECT was assessed in respect of 233 patients with known or suspected CAD. The 15.5 ± 4.9-year follow-up demonstrated that the overall outcome of these patients was favorable. Annualized event rates were relatively low during the entire follow-up period. Particularly, in the first 5 years after normal exercise ^99m^Tc-sestamibi myocardial perfusion SPECT, annualized event rates were very low. Predictors of increased risk were identified by multivariate analyses of clinical and exercise test data. Clinical predictors of adverse outcome were age, male gender, and diabetes. Exercise testing variables associated with an increased risk were heart rate at rest, peak exercise heart rate, diastolic blood pressure at rest, rate pressure product at rest, and ST segment changes.

Currently, there are no studies providing very long-term outcome data, and consequently the duration of the low-risk status after a normal test is not clear.^1^-^4^ Several previous studies have reported on the medium-term prognosis after a normal myocardial perfusion SPECT. In these previous studies, the number of included patients ranged from 88 to 273, and mean follow-up ranged from 10 months to 7.4 years.^1^-^3^ In the available literature, mean follow-up after myocardial perfusion SPECT was approximately 3 years.^1^-^3^ The previous studies have demonstrated that the medium-term prognosis of patients with suspected or known CAD and normal myocardial perfusion SPECT is favorable. Moreover, several large observational series have studied medium-term prognosis in patients with normal or low-risk thallium-201, ^99m^Tc-sestamibi, and ^99m^Tc-tetrofosmin. Meta-analyses of these series revealed that the annualized cardiac mortality rate was approximately 0.5%.^1^,^2^ The Dutch Heart Foundation data^8^ indicate that all-cause mortality in men aged 55-64 years, during the period 1986-1998 was (min-max) 2808-3175, and cardiac mortality was 206-422/100.000. In women aged 55-64 years, in the period 1985-1996, all-cause mortality was (min-max) 476-771, and cardiac mortality was 64.9-106/100.000. Hence, the event rates in the general population appear to be lower than in the study population. The study patients were referred to exercise myocardial perfusion SPECT by their treating physicians, and the risk profile of the patients was different from that of the general population. In the present study with 15-year follow-up annualized cardiac mortality rate was 0.3% using exercise ^99m^Tc-sestamibi myocardial perfusion SPECT.

The present study demonstrates that patients with a normal exercise ^99m^Tc-sestamibi myocardial perfusion SPECT have a favorable prognosis even at 15-year follow-up. In these patients a watchful waiting approach to care is justified, and additional diagnostic strategies including invasive coronary angiography can be avoided. Previous studies have shown that this type of management strategy may be both clinically effective and cost-effective.^9^,^10^ Clearly, clinical judgment remains important in deciding patient management decisions, also in patients with a normal exercise ^99m^Tc-sestamibi myocardial perfusion SPECT. The duration of the low-risk status depends on several factors that influence the natural progression of (subclinical) CAD. The present study demonstrates that several clinical and exercise test parameters can be used to identify patients at increased risk for adverse outcome. Prognosis was worse in patients with a history of CAD. Additionally multivariate analysis demonstrated that several clinical and exercise testing parameters influence very long-term outcome. Therefore, follow-up should be closer in patients with known CAD, and/or clinical and exercise parameters indicating higher risk status. Repeated testing should be considered in patients with a change in symptoms or worsening clinical status.

## Study Limitations

This study has some limitations. The study population and the number of adverse events were relatively small. Information on changes in medical therapy during follow-up was not available. During the period when the SPECT studies were performed, electrocardiogram gated acquisition was not routinely performed in our laboratory. Gated SPECT provides information on regional and global left ventricular function, which is an important predictor of long-term prognosis. Future studies are needed to clarify the value of gated SPECT for the assessment of very long-term prognosis in patients with normal myocardial perfusion. Finally, in the present study attenuation correction was not applied. Recent data indicate that attenuation correction may further improve diagnostic accuracy of myocardial perfusion SPECT.^11^,^12^

## Conclusion

Patients with suspected or known CAD and normal exercise ^99m^Tc-sestamibi myocardial perfusion SPECT have a favorable 15-year prognosis. Prognosis is particularly favorable during the initial 5 years after testing. Follow-up should be closer after this initial 5-year period and in patients with known CAD, and/or having clinical and exercise parameters indicating higher risk status.
